# Clinical Manifestations Laboratory Tests Abdominal Ultrasonic Findings and In-hospital Prognosis of COVID-19 in 185 Pediatric Cases in a Tertiary Center

**DOI:** 10.34172/aim.2023.100

**Published:** 2023-12-01

**Authors:** Maryam Jafari, Yasaman Mahalati, Elham Zarei, Mohammad Mahdi Kazemi, Arsalan Irompour, Amirhoessein Sadri, Hamed AzadiYekta

**Affiliations:** ^1^Department of Radiology Aliasghar Children’s Hospital School of Medicine Iran University of Medical Science Tehran Iran

**Keywords:** Abdominal findings, Children, COVID-19, Mortality, Prognosis, Ultrasound

## Abstract

**Background:**

Despite the COVID-19 pandemic, there is little information about the different clinical aspects of COVID-19 in children. In this study, we assessed the clinical manifestations, outcome, ultrasound, and laboratory findings of pediatric COVID-19.

**Methods:**

This retrospective study was conducted on 185 children with definitive diagnosis of COVID-19 between 2021 and 2022. The patients’ information was retrieved from hospital records.

**Results:**

The average age of the patients was 5.18 ± 4.55 years, and 61.1% were male. The most frequent clinical manifestation was fever (81.1%) followed by cough (31.9%), vomiting (20.0%), and diarrhea (20.0%). Mesenteric lymphadenitis was common on ultrasound and found in 60% of cases. In-hospital death was identified in 3.8% of cases. The mean length of hospital stay was 8.5 days. Mandating intensive care unit (ICU) stay was found in 19.5% and 5.9% of cases were intubated. Acute respiratory distress syndrome (ARDS), lower arterial oxygen saturation, higher white blood cell (WBC) count, and higher C-reactive protein (CRP) were the main determinants of death. Lower age, respiratory distress, early onset of clinical manifestations, lower arterial oxygen saturation, lower serum hemoglobin (Hb) level, and higher CRP level could predict requiring ICU admission.

**Conclusion:**

We recommend close monitoring on CRP, serum Hb level, WBC count, and arterial level of oxygenation as clinical indicators for potential progression to critical illness and severe disease. Mesenteric lymphadenitis is a common sonographic finding in pediatric COVID-19 which can cause abdominal pain. Ultrasound is helpful to avoid unnecessary surgical interventions in COVID-19.

## Introduction

As of late 2019, COVID-19 caused by a novel coronavirus first reported in China, has spread rapidly around the world. The clinical spectrum of COVID-19 varies widely from asymptomatic cases to acute respiratory distress syndrome and multi-organ involvement.^1-3^ On the other hand, there have always been concerns about the severity of COVID-19 in children and their risk of death.^4^ Some hypotheses were even formulated that perhaps the intensity of this condition in children is much lower than adults.^5^ In terms of epidemiology, about 18% of the total number of COVID-19 cases were related to children.^6,7^ In one study, about 75% of children suspected of the disease were seropositive for SARS-CoV-2.^8^ While the risk of developing the disease among children appeared to be much lower than among adults, the incidence of the disease in this age group was estimated to be similar to that of adults.^9^ According to some reports, the rate of seropositivity in suspected children was much higher than adults.^10^ Additionally, the rate of hospitalization with the severe form of COVID-19 in children ranged from 25.0 to 66.8 per 100 000 children in different age subgroups in one research.^11^ It should be kept in mind that the presence of underlying disorders could increase the need for hospitalization and intensive care unit (ICU) admission. According to the literature, age below one year was significantly associated with increasing hospitalization due to COVID-19.^12^ Unlike adults, confirmed death from COVID-19 is uncommon in children and adolescents, with an estimated rate of 0.17 per 100 000.^13,14^ Moreover, different studies have attempted to determine the host- and disease-related risk profiles for COVID-19 severity. Underlying conditions related to disease severity include medical complexity, underlying metabolic disturbances, neurological defects, obesity, and immunosuppressive state.^15-17^ According to the literature, most children suffering from severe COVID-19 had one or more underlying conditions.^18,19^ The clinical spectrum of COVID-19 in children ranges from asymptomatic to life-threatening states. Although about 15% to 42% of affected children remained asymptomatic,^20^ about 2% of them required mechanical ventilation and ICU admission.^21^ In general, to achieve a better outcome and prevent life-threatening complications in children with COVID-19, accurate assessment of clinical features, imaging and laboratory findings is necessary. In this study, in addition to detailed evaluation of clinical and paraclinical findings of children with COVID-19, we evaluate the clinical outcome of the patients as well as the relationship between the background findings and the prognosis of the disease in this age group.

## Materials and Methods

 This retrospective study was conducted on children with a definitive diagnosis of COVID-19 who were admitted to a referral children’s hospital in Tehran between April 2022 and May 2022. Of 875 cases with suspicious clinical findings of pediatric COVID-19, 185 patients met the inclusion criteria. We used convenience sampling to recruit participants. The most important factors for inclusion in this study were positive PCR tests and clinical manifestations of COVID-19; all laboratory data, abdominal ultrasound data, and follow-up data were available during their hospitalization. All suspected patients with clinical manifestations of COVID-19, such as fever, cough, respiratory distress, diarrhea, and vomiting, were assessed by reverse transcriptase-polymerase chain reaction (RT-PCR) and only those patients with a final positive test were analyzed. We collected the demographics, clinical characteristics, laboratory findings, and radiological findings from the hospital-recorded files and the hospital information system. Positive findings of the abdominal ultrasounds were classified. The clinical outcome was evaluated by follow-up of the hospitalized patients. In this regard, the in-hospital survival status and need for ICU admission were considered as prognostic criteria.

For statistical analysis, continuous variables were compared using the independent sample t-test or Mann-Whitney U-test whenever the data did not appear to have normal distribution or when the assumption of equal variances was violated across the study groups. In the independent sample t-test, the assumption of variance homogeneity was assessed to choose the best result and in the Mann-Whitney U test, the assumption of equal variances was not important. The chi-square/ Fisher’s exact test was used to compare the categorical variables according to the amount in the cross table. When more than 20% of cells had expected frequencies < 5, we used Fisher’s exact. *P* values of ≤ 0.05 were considered statistically significant. SPSS version 23.0 for Windows (IBM, Armonk, New York) was used.

## Results

In total, 185 children with COVID-19 were analyzed in this study. The demographic findings are summarized in Table 1. The average age of the patients was 5.18 ± 4.55 years. Considering gender, 61.1% were male. The most frequent clinical manifestation was fever (81.1%) followed by cough (31.9%), vomiting (20.0%), and diarrhea (20.0%). Regarding vital signs, the mean arterial oxygen saturation was 94.11 ± 3.56% and 13.5% had less than 90% saturation. Half of the affected children suffered from tachypnea.

**Table 1:** Patients’ demographic and clinical characteristics (n = 185)

**Characteristics**	**Number**
Mean age, year (Mean ± SD)	5.18 ± 4.55
Male gender, %	113 (61.1)
Exposure to other COVID-19 sources, %	86 (46.5)
Clinical manifestation, %	
Fever	150 (81.1)
Cough	59 (31.9)
Myalgia	13 (7.0)
Respiratory distress	35 (18.9)
Loss of consciousness	0 (0.0)
Anosmia	1 (0.5)
Taste disturbance	0 (0.0)
Seizure	1 (0.5)
Abdominal pain	9 (4.9)
Nausea	23 (12.4)
Vomiting	20.0)
Diarrhea	37 (20.0)
Disappetite	1 (0.5)
Headache	6 (3.2)
Average duration of symptoms, days (Mean ± SD)	2.97 ± 2.25
Clinical history, %	
History of cancer	4 (2.2)
History of diabetes mellitus	2 (1.1)
Hematological disorders	3 (1.6)
History of autoimmune disease	4 (2.2)
History of cardiac defects	1 (0.5)
History of renal disorder	9 (4.9)
History of dialysis	2 (1.1)
History of asthma	1 (0.5)
Vital signs	
Mean arterial oxygen saturation, (Mean ± SD)	94.11 ± 3.56
Respiratory rate, %	
14 to 18	47 (25.4)
19 to 22	49 (26.5)
23 to 28	48 (25.9)
> 28	41 (22.2)
Mean body temperature (Mean ± SD)	38.8 ± 1.37

On ultrasonographic assessment (Table 2), the positive findings were the following: increased renal echogenicity in 2.2% of cases, increased renal size in 4.9% of cases, mild bladder wall thickening in 1.1% of cases, sludge within the gallbladder in 3.8% of cases, splenomegaly in 8.1% of cases, and fatty liver in 5.4% of cases, pelvis fullness was detected in 3.2% with a mean anteroposterior diameter of 5 mm.

**Table 2:** The Laboratory Findings in of the Study Population (n = 185)

**Laboratory Parameters**	**Number **
Mean hemoglobin level, g/dL (Mean ± SD)	10.05 ± 1.95
Mean white blood cell count, /mm^3^ (Mean ± SD)	10.55 ± 6.51
Mean platelet count, /mm^3^ (Mean ± SD)	247.43 ± 26.61
Mean CRP (Mean ± SD)	59.55 ± 33.26
Mean ESR (Mean ± SD)	39.03 ± 24.10
Mean HCO_3_, mEq/L (Mean ± SD)	18.70 ± 6.02
Mean PCO_2_, mm Hg (Mean ± SD)	29.80 ± 9.14
Mean pH (Mean ± SD)	7.41 ± 0.12
Raised glucose level (%)	10 (5.4 )
Raised BUN (%)	5 (2.7)
Raised creatinine (%)	4 (2.2)
Raised AST (%)	5 (2.7)
Raised ALT (%)	7 (3.8)
Raised LDH (%)	4 (2.2)

ICU, intensive care unit; CRP, C-reactive protein; AST, Aspartate transaminase; ALT, alanine transaminase; LDH, lactate dehydrogenase; BUN, blood urea nitrogen; ESR, erythrocyte sedimentation rate.

**Figure 1:**
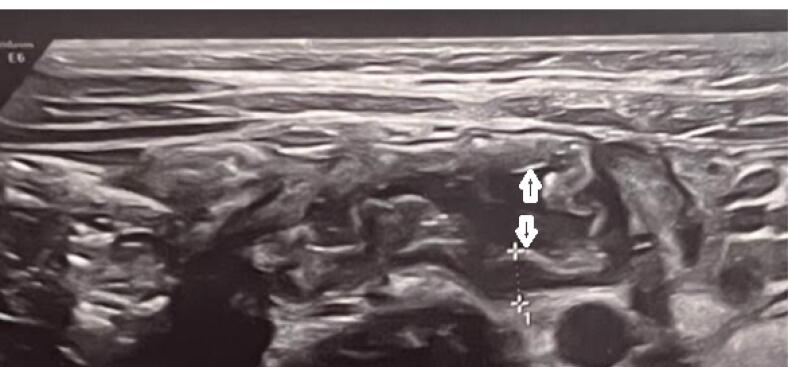
Ultrasound image demonstrates evidence of terminal ileitis in a 4-year-old girl with COVID disease. Gray-scale US shows the thickened terminal ileum, wall thickness (white calipers) and lumen (double-headed arrow)

Enlarged mesenteric lymph nodes were found on ultrasonography in 32.4% of cases; cases with three or more nodes with a short-axis diameter of at least 5 mm clustered in the right lower quadrant were considered positive. The ​​enlarged lymph nodes were located anterior to the right psoas muscle and in the small bowel mesentery. In 7 % of cases, the terminal ileum was thick-walled with a single layer of over 3mm (Figure 1). Evidence of pleural effusion was found in 2.2% of cases. Interestingly, appendicitis and appendectomy were found in 2.7%.

 Considering disease outcome, in-hospital death was found in 3.8% of cases. The mean length of hospital stay was 8.5 days. Need for ICU admission was found in 19.5%. Intubation was performed in 5.9% of cases. Of those who did not survive, four patients suffered from abdominal ascites, fatty liver, and raised liver enzymes, two patients had gallbladder abnormalities, and one had renal disorders requiring hemodialysis.

Comparing baseline information between non-surviving and surviving subjects (Table 3), the non-surviving showed a higher prevalence rate of respiratory distress (85.7% versus 16.5%, *P* < 0.001, odds ratio [OR] > 1), significantly lower mean arterial oxygen saturation (86.33 ± 5.95% versus 94.38 ± 3.14%, *P* < 0.001, OR < 1), higher mean white blood cell (WBC) count (18.65 ± 4.03 versus 9.78 ± 6.22, *P* = 0.044, OR > 1), and higher mean C-reactive protein (CRP) (60.41 ± 33.49 versus 33.12 ± 11.29, *P* = 0.002). Comparing subgroups with and without ICU admission (Tables 4 and 5), younger age (*P* = 0.018), respiratory distress (*P* < 0.001, OR > 1), early onset of clinical symptoms (*P* = 0.032), lower arterial oxygen saturation (*P* < 0.001, OR < 1), tachypnea (*P* < 0.001, OR > 1), lower serum hemoglobin (Hb) level (*P* = 0.001, OR < 1) and higher CRP level (*P* = 0.010) were significantly different as the main determinants of requiring ICU admission. Tables 6 and 7 provide further details.

**Table 3:** Ultrasound Findings of the Study Population (n = 185)

**Ultrasound Findings**	**Number (%)**
Pelvic fullness	
Normal	133 (71.9)
Fullness	46 (24.9)
Hydronephrosis	6 (3.2)
Renal echogenicity	
Normal	180 (97.3)
Increased	4 (2.2)
Decreased	1 (0.5)
Renal size	
Normal	173 (93.5)
Increased	9 (4.9)
Decreased	3 (1.6)
Bladder wall status	
Normal	181 (97.8)
Increased	2 (1.1)
Decreased	2 (1.1)
Pancreas echogenicity	
Normal	184 (99.5)
Increased	1 (0.5)
Gall bladder status	
Normal	149 (80.5)
Contracted	29 (15.7)
Sludge	7 (3.8)
Spleen status	
Normal	170 (91.9)
Splenomegaly	15 (8.1)
Increased liver echogenicity	10 (5.4)
Liver size	
Normal	184 (99.5)
Increased	1 (0.5)
Appendicitis	5 (2.7)
Large mesenteric lymph nodes	60 (32.4)
Bowel status	
Normal	
Increases wall thickness (FIG 1)	13 (7)
Abdominal mass	4 (2.2)
Pleural effusion (mild)	5 (2.7)

**Table 4:** Baseline Characteristics Based on Survival Status (n = 185)

**Baseline Characteristics**	**Non-surviving** **(n=7)**	**Surviving** **(n=176)**	***P*** ** Value**
Mean age, year	3.57 ± 1.87	5.24 ± 0.34	0.342
Male gender, No. (%)	5 (71.4)	108 (61.4)	0.591
Exposure to COVID-19 cases, No. (%)	3 (42.9)	83 (47.2)	0.823
Clinical manifestation, No. (%)			
Fever	4 (57.1)	146 (83.0)	0.112
Cough	1 (14.3)	58 (33.0)	0.432
Myalgia	0 (0.0)	13 (7.4)	0.456
Respiratory distress	6 (85.7)	29 (16.5)	< 0.001
Anosmia	0 (0.0)	1 (0.6)	0.999
Seizure	0 (0.0)	1 (0.6)	0.999
Abdominal pain	0 (0.0)	9 (5.1)	0.539
Nausea	1 (14.3)	22 (12.5)	0.889
Vomiting	1 (14.3)	36 (20.5)	0.690
Diarrhea	1 (14.3)	36 (20.5)	0.690
Disappetite	0 (0.0)	1 (0.6)	0.999
Headache	1 (14.3)	5 (2.8)	0.211
Average duration of symptoms, days	3.00 ± 2.44	2.97 ± 2.25	0.975
Clinical history, No. (%)			
History of cancer	0 (0.0)	4 (2.3)	0.687
History of diabetes mellitus	0 (0.0)	2 (1.1)	0.777
Hematological disorders	0 (0.0)	3 (1.7)	0.728
History of autoimmune disease	0 (0.0)	4 (2.3)	0.687
History of cardiac defects	1 (14.3)	0 (0.0)	0.038
History of renal disorder	0 (0.0)	9 (5.1)	0.539
History of dialysis	0 (0.0)	2 (1.1)	0.777
History of asthma	0 (0.0)	1 (0.6)	0.999
History of other respiratory disease	0 (0.0)	1 (0.6)	0.999
Vital signs			
Mean arterial oxygen saturation, No. (%)	86.33 ± 5.95	94.38 ± 3.14	< 0.001
Respiratory rate, No. (%)			0.327
14 to 18	1 (14.3)	46 (26.1)	
19 to 22	2 (28.6)	54 (30.7)	
23 to 28	1 (14.3)	47 (26.7)	
> 28	3 (42.9)	29 (16.5)	
Mean body temperature	38.00 ± 0.94	38.08 ± 1.39	0.883
Laboratory parameters			
Mean hemoglobin level, g/dL	8.70 ± 2.55	10.18 ± 1.86	0.186
Mean white blood cell count, /mm^3^	18.65 ± 4.03	9.78 ± 6.22	0.044
Mean platelet count, /mm^3^	230.25 ± 189.62	251.47 ± 58.33	0.888
Mean CRP	60.41 ± 33.49	33.12 ± 11.29	0.002
Mean ESR	45.67 ± 37.42	38.79 ± 23.61	0.646
Mean HCO_3_, mEq/L	15.00 ± 3.67	18.82 ± 6.15	0.394
Mean PCO_2_, mm Hg	35.94 ± 18.15	28.89 ± 7.04	0.108
Mean pH	7.25 ± 0.01	7.41 ± 0.02	0.209
Raised glucose level, No. (%)	0 (0.0)	10 (5.7)	0.517
Raised BUN, No. (%)	0 (0.0)	5 (2.8)	0.651
Raised creatinine, No. (%)	0 (0.0)	4 (2.3)	0.687
Raised AST, No. (%)	0 (0.0)	5 (2.8)	0.651
Raised ALT, No. (%)	0 (0.0)	7 (4.0)	0.591
Raised LDH, No. (%)	0 (0.0)	4 (2.3)	0.687
Raised ALP, No. (%)	0 (0.0)	6 (3.4)	0.619

CRP, C-reactive protein; AST, Aspartate transaminase; ALT, alanine transaminase; LDH, lactate dehydrogenase; BUN, blood urea nitrogen; ESR, erythrocyte sedimentation rate.

**Table 5:** Baseline Characteristics Based on ICU Status (n = 185)

**Baseline Characteristics**	**ICU Admission (+)** **(n=36)**	**ICU Admission (-)** **(n=147)**	***P*** ** Value**
Mean age, year	3.69 ± 3.92	5.54 ± 4.63	0.018
Male gender, No. (%)	20 (55.6)	93 (63.3)	0.394
Exposure to COVID-19 cases, No. (%)	15 (41.7)	71 (48.3)	0.475
Clinical manifestation, No. (%)			
Fever	26 (72.2)	124 (84.4)	0.090
Cough	10 (27.8)	49 (33.3)	0.523
Myalgia	1 (2.8)	12 (8.2)	0.260
Respiratory distress	21 (58.3)	14 (9.5)	< 0.001
Anosmia	0 (0.0)	1 (0.7)	0.999
Seizure	1 (2.8)	0 (0.0)	0.197
Abdominal pain	0 (0.0)	9 (6.1)	0.209
Nausea	4 (11.1)	19 (12.9)	0.789
Vomiting	5 (13.9)	32 (21.8)	0.291
Diarrhea	4 (11.1)	33 (22.4)	0.129
Disappetite	0 (0.0)	1 (0.7)	0.999
Headache	1 (2.8)	5 (3.4)	0.851
Average duration of symptoms, days	2.42 ± 1.34	3.10 ± 2.40	0.032
Clinical history, No. (%)			
History of cancer	0 (0.0)	4 (2.7)	0.317
History of diabetes mellitus	1 (2.8)	1 (0.7)	0.278
Hematological disorders	1 (2.8)	2 (1.4)	0.548
History of autoimmune disease	1 (2.8)	3 (2.0)	0.786
History of cardiac defects	1 (2.8)	0 (0.0)	0.197
History of renal disorder	2 (5.6)	7 (4.8)	0.691
History of dialysis	1 (2.8)	1 (0.7)	0.278
History of asthma	0 (0.0)	1 (0.7)	0.999
History of other respiratory disease	0 (0.0)	1 (0.7)	0.999
Vital signs			
Mean arterial oxygen saturation, Mean ± SD	90.33 ± 5.27	94.98 ± 2.30	< 0.001
Respiratory rate, No. (%)			0.001
14 to 18	7 (19.4)	40 (27.0)	
19 to 22	11 (30.6)	46 (31.1)	
23 to 28	4 (11.1)	44 (29.7)	
> 28	14 (38.9)	18 (22.2)	
Mean body temperature	37.94 ± 0.75	38.11 ± 1.48	0.520
Laboratory parameters			
Mean hemoglobin level, g/dL	8.85 ± 2.48	10.72 ± 1.16	0.001
Mean white blood cell count, /mm^3^	13.60 ± 9.19	10.10 ± 6.21	0.398
Mean platelet count, /mm^3^	320.14 ± 125.82	211.07 ± 59.21	0.379
Mean CRP	105.23 ± 11.17	54.66 ± 31.04	0.010
Mean ESR	40.00 ± 21.72	38.67 ± 25.31	0.884
Mean HCO_3_, mEq/L	17.88 ± 5.00	18.83 ± 6.23	0.725
Mean PCO_2_, mm Hg	29.51 ± 11.48	29.98 ± 7.59	0.880
Mean pH	7.40 ± 0.16	7.41 ± 0.09	0.755
Raised glucose level, No. (%)	5 (13.9)	5 (3.4)	0.013
Raised BUN, No. (%)	2 (5.6)	3 (2.0)	0.253
Raised creatinine, No. (%)	1 (2.8)	3 (2.0)	0.782
Raised AST, No. (%)	0 (0.0)	5 (3.4)	0.585
Raised ALT, No. (%)	1 (2.8)	6 (4.1)	0.720
Raised LDH, No. (%)	0 (0.0)	4 (2.7)	0.319
Raised ALP, No. (%)	2 (5.6)	4 (2.7)	0.334

ICU, intensive care unit; CRP, C-reactive protein; AST, Aspartate transaminase; ALT, alanine transaminase; LDH, lactate dehydrogenase; BUN, blood urea nitrogen; ESR, erythrocyte sedimentation rate.

**Table 6:** Comparison of Significant Clinical and Laboratory Findings based on Survival Status

**Variable**	***P*** ** value**	**OR**	**95% CI**
Respiratory distress	0.002	30.414	3.528-262.18
Arterial oxygen saturation	0.002	0.885	0.727-1.075
WBC	0.005	0.944	0.785-1.134
CRP	0.013	3.545	0.668-18.819

WBC, white blood cell; CRP, C-reactive protein.

**Table 7:** Comparison of Significant Demographic, Clinical and Laboratory Characteristics Based on ICU Status

**Variable**	***P*** ** value**	**OR**	**95% CI**
Age	0.016	0.451	0.245-0.868
Respiratory distress	0.000	10.833	4.692-25.014
Duration of clinical symptoms	0.014	0.827	0.643-1.065
Arterial oxygen saturation	0.000	0.782	0.531-0.982
Tachypnea	0.009	1.351	0.953-1.916
Serum hemoglobin	0.005	10.785	6.662-15.931
CRP	0.004	0.777	0.368-1.641

ICU, intensive care unit; CRP, C-reactive protein.

## Discussion

This study aimed to evaluate the epidemiological, clinical, diagnostic, and prognostic aspects of COVID-19 in children. Also, a comprehensive evaluation of abdominal ultrasound data was undertaken to investigate the ultrasound findings of pediatric COVID-19. Our study showed that boys were more involved. Also, the evaluation of the age curve of the patients showed a high prevalence of hospitalization in children under 5 years, as 62% of the affected children were five years old or younger. This finding showed little difference from other similar studies in other communities. In a study by Karbuz et al^22^ in Turkey, the majority of patients were between 6 and 12 years of age. In a review by Ding et al, the majority of pediatric patients with COVID-19 were older than 5 years of age^23^ which is consistent with our study. In another study among Iranian pediatric patients, similar to our report, 60.4% were male while their mean age was 32 months.^24^

Regarding history of exposure to COVID-19, 46.5% of our patients reported such exposure. The history of exposure to COVID-19 was recorded in 75.2% of cases in the study by Karbuz et al,^22^ 75.6% in a study by Hoang et al,^25^ and 86.4% in the study by Ding et al.^26^ It seems that the families in Iran are much more sensitive in decreasing the exposure of their children to the sources of the spread of the virus in the society. Social distancing has been implemented more favorably in this society.

Regarding the presence of underlying conditions, few of our children (below 5%) had predisposing and underlying diseases which was notably higher in other reports such as CDC-MMWR. According to the CDC-MMWR, 23% of pediatric patients had underlying conditions. The most common underlying conditions were chronic lung disease (including asthma) followed by cardiovascular disease and immunosuppression.^27^ In another report, 6.1% of all the included children had underlying diseases.^25^ According to another research, the most common underlying medical conditions were history of immunosuppression and history of respiratory or cardiac disease (65%).^26^ In our study, the most common underlying disorder was history of chronic renal disease found in only 4.9%. In other reports, lung disorders and congenital heart disease were the common underlying conditions.^26,27^ A history of asthma was found in only one patient in our study, whereas in some reports, simultaneous history of asthma has been suggested as a risk factor for COVID-19 occurrence and severity.^28-30^ Among the common symptoms, the most prevalent presentations were fever and cough, similar to the literature.^22-25,31^ In laboratory parameters, in our sample, the two prominent laboratory findings were leukocytosis and high CRP level. They were also the main determinants for poorer prognosis. In other reports, the main laboratory findings were leukopenia, leukocytosis, increased creatine kinase, and thrombocytopenia.

**Figure 2:**
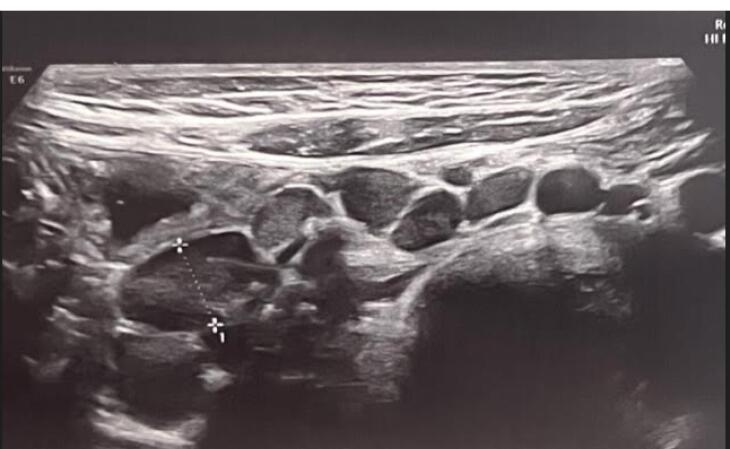
Ultrasound Image Depicting Multiple Reactive Mesenteric Lymph Nodes with a Preserved Oval-Shape and Intact Outline (Mesenteric Lymphadenitis) Anterior to the Psoas Muscle in the Right Lower Quadrant in a 4-Year-Old Girl with COVID Disease

One of the main findings on abdominopelvic ultrasound was common involvement of some abdominal organs including mesenteric lymph nodes and splenomegaly. According to the studies, involvement of these organs has always been an indication of the severity of the disease, especially in the context of multi-system inflammatory syndrome (MISC) in children with COVID-19.^32^ Mesenteric lymphadenitis (Figures 1 and 2) and splenomegaly were the most common findings in ultrasound. According to the previous studies, these findings have been the signs of the severity of COVID-19, especially in the context of MISC syndrome in children with COVID-19. MIS-C, first reported in April 2020, is currently a post-COVID-19 complication and could be related to the immune response to infection.^32^ Gastrointestinal symptoms are most significant in MIS-C, occurring in about 92% of cases, followed by cardiac involvement in about 80%.^33,34^ These GI symptoms can mimic many other infections and inflammatory conditions in children, including the acute abdomen, as in our cases with appendicitis.^32-37^

As in our study, mesenteric lymphadenitis was the most common abdominal finding in 60% of cases with COVID-19 disease which was similar to previous studies. Considering previous studies, it has been shown that imaging studies, such as abdominal ultrasound or even abdominal computed tomography (CT), may simplify differentiating true surgical emergencies in questioned cases of the acute abdomen.^34^ In our study, abdominal ultrasound was practical in cases with abdominal pain, and similar to other studies, surgically confirmed cases of acute appendicitis were found in patients with COVID-19 in our investigation.^38-42^ However, in a few studies, non-operative management of uncomplicated acute appendicitis accompanying COVID-19 infection with antibiotics has been documented.^43^

 There is an association between the severity of COVID-19 and involvement of abdominal organs, including enlarged mesenteric lymph nodes, kidney involvement leading to dialysis, and liver involvement leading to increased liver enzymes and fatty liver disease. Therefore, ultrasound evidence related to the involvement of these organs can be also considered as a prognostic factor.

 Our study has a variety of limitations to consider as it was conducted in a single-center pediatric department, in addition to the limitations of any retrospective analysis. Our research was conducted in a different region of the world compared to previous studies, and more so, a distinct region of Iran, which is another limitation of this study. Additionally, a broader period of data collection and larger sample sizes may have further demonstrated more cases of severe presentations, more complications, and favorable findings. Finally, the number of non-surviving cases in our study was seven cases which is one of the important limitations of our study.

## Conclusion

Regarding prognostic factors, some baseline parameters including younger age, lower arterial oxygen saturation, and evidence of acute respiratory distress, raised CRP, and lowering Hb level could effectively predict poorer clinical outcomes including death and ICU requirement. A recent meta-analysis found acute respiratory distress syndrome and acute kidney injury as factors predicting admission to ICU; and shortness of breath, neurological symptoms, raised CRP level, and D-dimer level as predicting factors for progression to severe or critical disease and death.^44^ These factors will be useful for designing models predicting hospital and long-term outcomes of children with COVID-19.

 In general, the mortality rate of these children is still low and need for special care is raised in the minority of these patients. It seems that Iran has been very successful in managing such patients.
